# Process evaluation of an implementation intervention to facilitate the use of the Swedish Physical Activity on Prescription in primary healthcare

**DOI:** 10.1186/s12913-023-09974-8

**Published:** 2023-09-15

**Authors:** Catharina Gustavsson, Maria Nordqvist, Åsa Bergman Bruhn, Kristina Bröms, Lars Jerdén, Lena V. Kallings, Lars Wallin

**Affiliations:** 1 Center for Clinical Research Dalarna, Uppsala University, Nissers Vag 3, 79182 Falun, Sweden; 2 School of Health and Welfare, Dalarna University, 79188 Falun, Sweden; 3 Department of Public Health and Caring Sciences, Uppsala University, BMC, Box 564, 751 22 Uppsala, Sweden; 4 Department of Physical Activity and Health, Swedish School of Sport and Health Sciences (GIH), Box 5626, 114 86 Stockholm, Sweden

**Keywords:** Disease prevention, Exercise, Health professionals, Health promotion, Implementation, Mixed methods research

## Abstract

**Background:**

The Swedish Physical Activity on Prescription (PAP-S) is a method for healthcare to promote physical activity for prevention and treatment of health disorders. Despite scientific support and education campaigns, the use has been low. The aim of this study was to perform a process evaluation of an implementation intervention targeting the use of the PAP-S method in primary healthcare (PHC). Specifically, we wanted to evaluate feasibility of the implementation intervention, and its effect on the implementation process and the outcome (number of PAP-S prescriptions).

**Methods:**

This was a longitudinal study using the Medical Research Council guidance for process evaluation of a 9-month implementation intervention among healthcare staff at three PHC centres in Sweden. Data was collected by: participatory observations of the implementation process; questionnaires to the staff before, after and 6 months after the implementation intervention; interviews after the implementation intervention; and number of PAP-S prescriptions.

**Results:**

During the implementation intervention, the workplaces’ readiness-to-change and the healthcare staff’s confidence in using the PAP-S method were favourably influenced, as was the number of PAP-S prescriptions. After the implementation intervention, the number of PAP-S prescriptions decreased to about the same number as before the implementation intervention, at two out of three PHC centres. Four of the six implementation strategies appeared to impact on the implementation process: external facilitation; leadership engagement by a committed workplace management; local PAP-S coordinator taking a leading role and acting as local champion; educational outreach concerning how to use the PAP-S method.

**Conclusion:**

The implementation intervention was not sufficient to produce sustained change of the healthcare staff’s behaviour, nor did it achieve favourable long-term outcome on the number of PAP-S prescriptions. The healthcare staffs’ sparse knowledge of the PAP-S method prior to the implementation intervention hampered the implementation. More hands-on education in how to use the PAP-S method introduced early in the implementation process is imperative for successful implementation of the PAP-S method. The findings also suggest that committed workplace management and local PAP-S coordinators, taking leading roles and acting as local champions, need to be firmly established at the PHC centres before the external facilitator withdraws.

**Trial registration:**

Registered in the ISRCTN registry with study registration number: ISRCTN15551042 (Registration date: 12/01/2016).

**Supplementary Information:**

The online version contains supplementary material available at 10.1186/s12913-023-09974-8.

## Background

Physical activity has a favourable impact on mental and physical health [1]. Physical inactivity is an important risk factor for loss of disability-adjusted life years [2]. Healthcare services have an important role in promoting healthy physical activity behaviours, since they reach large parts of the population, and have substantial credibility in regard to conveying health information [3]. In 2011, the Swedish National Board of Health and Welfare launched guidelines for healthcare methods in the prevention of diseases due to unhealthy lifestyle [4] which were updated in 2018 [5]. Regarding insufficient physical activity, these guidelines recommend that healthcare providers should offer person-centred health promotion counselling, which could be combined with a written prescription of physical activity and monitoring of physical activity.

The Swedish Physical Activity on Prescription (PAP-S) was launched in 2001, as a method for healthcare providers to promote physical activity for the prevention and treatment of health disorders [6]. Studies have reported that PAP-S increases physical activity in patients [7–9], reduces sedentary time [10], increases health-related quality of life [7, 11, 12] and reduces risk factors for metabolic syndrome and cardiovascular diseases [10, 13]. Despite scientific support for PAP-S and major information and education campaigns aimed at healthcare professionals, the method is not widely used in healthcare [4, 14, 15]. In a previous interview study we reported that healthcare professionals acknowledged the importance of promoting physical activity in patient encounters, but claimed that they lacked time, written routines and in some cases competence in using PAP-S for that purpose [16]. Another study also showed a gap between how highly healthcare professionals ranked the importance of promoting physical activity, versus how much they actually promoted it during patient consultations [17]. Both the World Health Organization (WHO) and the European Commission have declared PAP-S as best practice to be implemented in European Union Member States [18], but there is insufficient knowledge on how to support implementation of PAP-S in healthcare [19–21]. In summary, although the PAP-S method has substantial scientific support of beneficial health effects and that healthcare professionals acknowledge the importance of promoting physical activity in patient consultations, attempts to support the use of the method in healthcare have not yielded lasting effects on behaviour change. There is clearly a need for studies that explore how implementation of the PAP-S method can be supported.

Implementation is defined as a specified set of activities designed to put into practice a specific program or method of known dimensions [22]. (Explanation of key terms relating to implementation is outlined in Table 1). When assessing a new method, such as the PAP-S method, it is important to distinguish between evaluating the effects of the method and the implementation of the method [23]. Implementation research focuses on the implementation process, how it can be supported and the factors affecting it. This means that implementation interventions comprise a bundle of implementation strategies, designed to change behaviours at organisational, practitioner, or patient level [24, 25]. Implementation strategies are defined as the discrete activities or techniques used to enhance the adoption, implementation, and sustainability of an innovation/method/practice, such as the PAP-S method [26]. Implementation of new methods in healthcare involves a process of behavioural change, both at the individual and organisational level, and is affected by several factors [24, 27]. Health promotion methods often take longer to implement in clinical practice as compared to the introduction of new technologies [24]. The Cochrane Effective Practice and Organisation of Care (EPOC) group classifies barriers to professional and organisational behaviour change in healthcare into nine categories: information management, clinical uncertainty, sense of competence, perceptions of liability, patient expectations, standards of practice, financial disincentives, administrative constraints, and other barriers [28]. The framework integrated Promoting Action on Research Implementation in Health Services (i-PARIHS) describes successful implementation as a function of the interplay between the innovation/method/practice to be implemented, the recipients who will use and/or are targeted by the innovation/method/practice, and the context in which it is to be implemented [29]. Facilitation activates the implementation by responding to, and supporting, the features of the proposed innovation/method/practice [30].

**Table 1:** Overview and explanations of key terms in the study

Key terms	Explanation of key terms
Implementation	A specified set of steps and activities designed to put into practice a specific intervention, program or method of known dimensions [22]
Implementation research	Systematic and scientific studies that focuses on understanding and improving the processes involved in the implementation of interventions, programs, or methods in real-world settings. It investigates the barriers and facilitators to successful implementation, explores how to adopt interventions to different contexts, and identifies strategies to enhance uptake and sustainability of evidence-based practices. The goal of implementation research is to promote effective translation of research findings into actions that lead to positive outcomes in for example healthcare practice [22, 28, 30]
Implementation intervention	A specific action or approach designed to facilitate and support the successful implementation of an innovation/method/practice. It is designed to change behaviours at organisational, practitioner, or patient level, and aims to address the barriers and challenges that may arise during the implementation process and improve adoption and integration of the proposed changes into the targeted setting. Implementation interventions can take various forms, such as training and capacity building, stakeholder engagement activities, using evidence-based implementation strategies etcetera. The selection of appropriate implementation intervention depends on the context, the nature of the innovation/method/practice to be implemented and the specific challenges that need to be addressed [22, 24, 25]
Implementation process	Execution of the activities, steps and tasks outlined in the implementation plan, including allocating resources, assigning responsibilities and managing the necessary activities to achieve the intended goal [22, 29, 31, 32]
Implementation strategies	Discrete activities or techniques used to enhance the successful adoption, integration, and sustainability of an innovation/method/practice in a specific setting or context. These strategies are designed to overcome barriers, enhance the implementation process and promote the uptake of evidence-based practices. Implementation strategies can be diverse and tailored to the unique needs of the situation. The selection of appropriate implementation strategies depends on several factors, including the nature of the innovation/method/practice, the context, the available resources, and potential barriers to implementation [26, 28, 33]
Implementation activities	Specific actions undertaken to put a method (innovation, program etc.) into practice. Implementation activities are part of the implementation process and involve executing the strategies and other steps outlined in the implementation plan [22, 28, 29]
Process evaluation	Method to assess the implementation and execution of an intervention. That is, it focuses on assessment of the process of an intervention instead of the effect of an intervention. It involves examining the procedures, activities, and resources used to deliver the intervention and how well they align with the intended objectives. A process evaluation helps to identify strengths, weaknesses and areas for improvements, which contributes to a better understanding of how and why an intervention is effective or requires adjustments [31, 32, 34]

A range of strategies to support implementation have been described [33] and systematic reviews suggest that many strategies have a similar moderate effect on implementation outcomes [28]. The impact of implementation strategies involving educational outreach, reminder systems, and audit and feedback, was shown to be between 4–6% of improvement. The use of so-called ‘opinion leaders’ was the strategy that showed the best effect: a 12% median effect improvement [28]. Implementation interventions using a multi-faceted approach, i.e. concurrent use of multiple strategies to support implementation, has been suggested favourable when compared to using single strategies [27]. It has also been emphasised that implementation strategies should be tailored to local conditions in order to fit in as part of regular procedures in the specific context [35] and that engagement of the local leadership is important for successful implementation [36].

The Medical Research Council’s (MRC) guidelines for complex interventions recognises the value of undertaking a process evaluation to assess the quality of implementation, and causal mechanisms and contextual factors associated with outcomes [34]. The MRC has also provided guidance for carrying out a process evaluation, built on describing what is implemented and how, exploring mechanisms that produce change, and how the context affects implementation [31].

The aim of the study was to perform a process evaluation of an implementation intervention targeting the use of the PAP-S method in primary healthcare (PHC). Specifically, we wanted to evaluate feasibility of the implementation intervention, and its effect on the implementation process and the outcome (number of PAP-S prescriptions).

## Materials and methods

### Study design

**Fig. 1:**
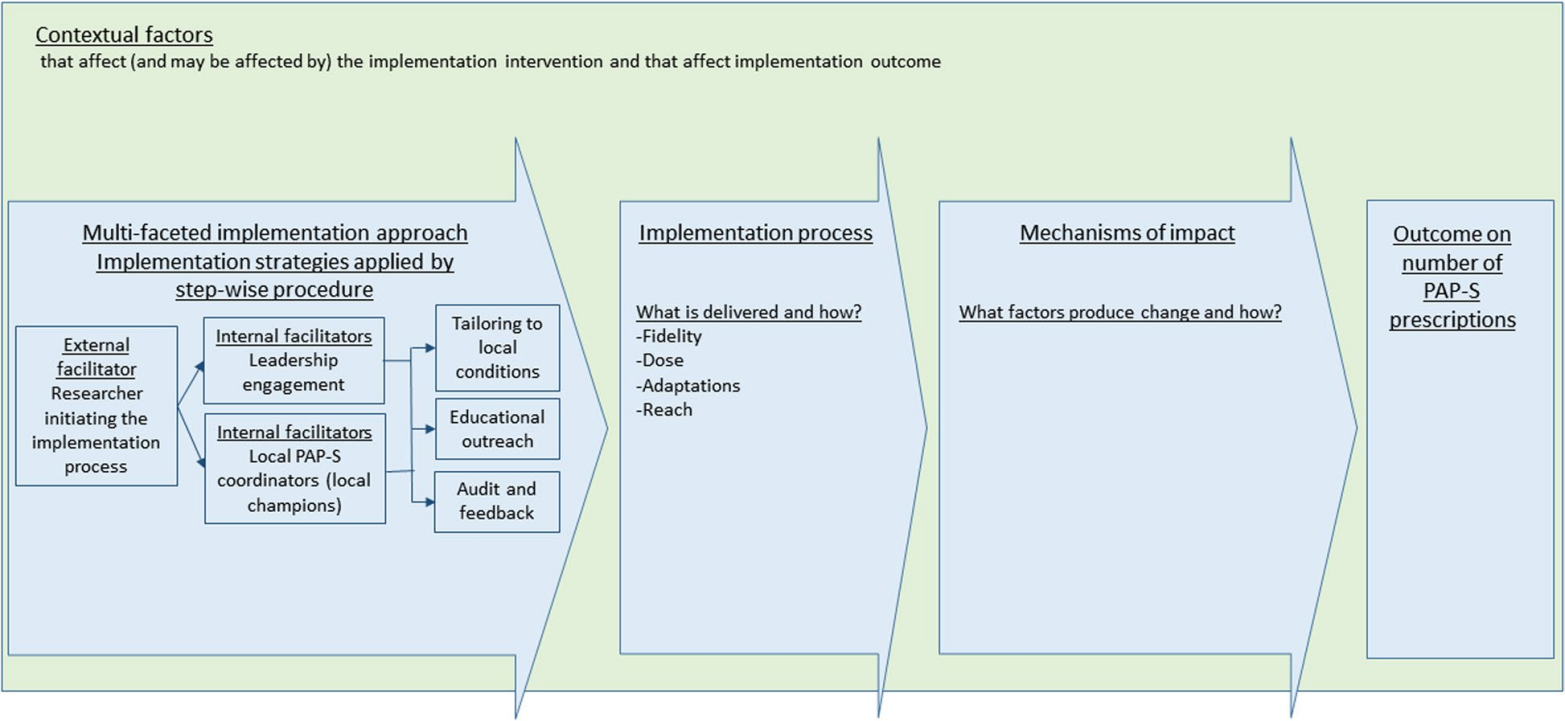
Outline of methods for data collection for process evaluation according to the Medical Research Council guidance

**Fig. 2:**
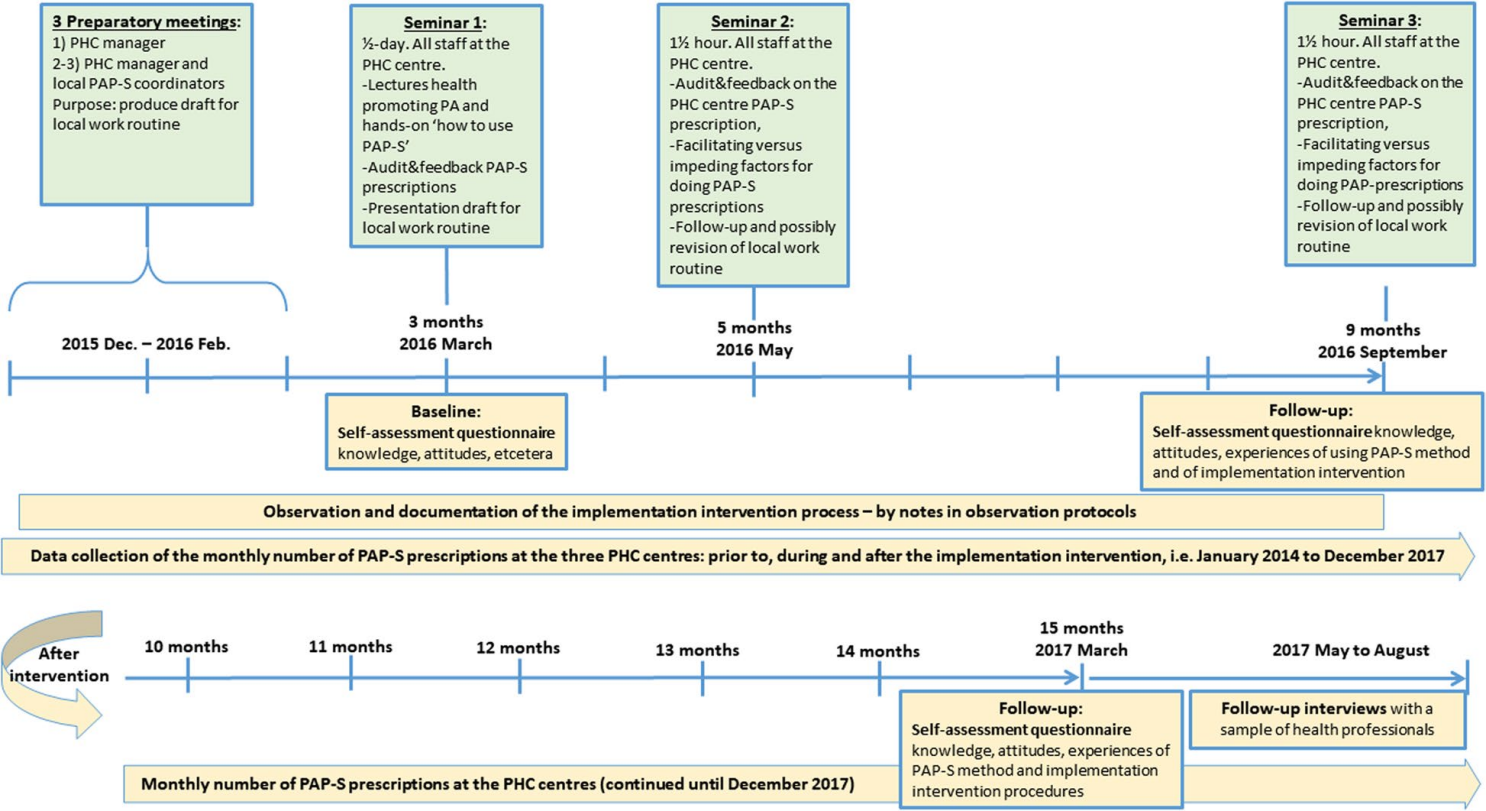
Overview of procedures in the 9 months implementation intervention and follow-ups

We conducted a longitudinal process evaluation [31] of an implementation intervention [25] guided by the MRC framework [31] (Fig. 1), focusing on describing what is implemented and how, the mechanisms of impact that produce change, and how context affected implementation. A mixed methods approach with a convergent design [37] was employed in that data was collected by both qualitative [38] (i.e. observations and interviews) and quantitative [39] (i.e. aggregated healthcare statistics and questionnaires) methods (Fig. 2). The qualitative and quantitative data were analysed separately and were then merged in the interpretation of results.

### Context and participants

The PHC centres were selected by a purposeful sampling approach [40], which was considered relevant for this process evaluation. Thus, no a priori power calculation was conducted. The sampling approach aimed to achieve inclusion of PHC centres that represented a purposeful variation in contextual/organisational characteristics relevant to Swedish PHC. Three PHC centres in two regional healthcare organisations were chosen: one large PHC centre situated in a blue-collar workers’ area in a mid-sized town (PHC-C1), one small PHC centre in a rural farming area (PHC-C2) and one mid-sized PHC centre in a small blue-collar workers’ town (PHC-C3). All PHC centres were financed by the public healthcare system, but PHC-C3 was privately managed. All healthcare professionals who had patient consultations and the management at the three PHC centres were invited to participate. Verbal and written information about the study was provided, and written consent to participate was obtained from the participants prior to the intervention. All healthcare professionals, except medical secretaries and short-term employed physicians, participated: 41 at PHC-C1; 17 at PHC-C2; and 18 at PHC-C3.

### Procedure

The 9-month implementation intervention took place at each PHC centre from December 2015 to middle of September 2016. The study procedures, time points for meetings and seminars that were part of the implementation intervention, and follow-ups, are displayed in Fig. 2. All participants completed a questionnaire at baseline, i.e. prior to attending their first meeting. The meetings and seminars were monitored by participatory observations [40]. Follow-up was undertaken by: 1) questionnaires to all participants at the end of the implementation intervention (September 2016) and at six months after it ended (March 2017); 2) interviews with a sample of the participants (during the summer 2017); and 3) statistics on monthly number of PAP-S prescriptions at each PHC centre collected prior to, during and after the implementation intervention (January 2014 to December 2017).

### The clinical intervention – PAP-S

The PAP-S is a method for healthcare providers to promote physical activity for the prevention and treatment of health disorders [6]. All licensed healthcare professions with sufficient knowledge of the PAP-S method can use PAP-S in patient consultations. The PAP-S method consists of five core components: 1) a person-centred health promotion consultation, 2) prescription of physical activity based on the patient’s goals which is detailed in a written agreement between the patient and the healthcare professional, 3) the prescription should be guided by evidence-based physical activity recommendations (e.g. by the handbook ‘FYSS—Physical Activity in the Prevention and Treatment of Disease’ [41, 42]), 4) planned follow-up of the prescription, and 5) collaboration with physical activity organisers outside healthcare (e.g. sports clubs, fitness centres). It is emphasised that the PAP-S method should be tailored to the local conditions in the specific healthcare organisation [14, 43].

### Implementation intervention

The i-PARIHS framework [29] guided the planning of the implementation intervention which aimed to support healthcare professionals in the use of the PAP-S method for promoting physical activity in patient consultations in their clinical practice. The implementation intervention consisted of a multi-faceted approach entailing a bundle of implementation strategies [33]:

- *External facilitation* [30], one of the researchers (first author) acted as an *external facilitator*, activating the implementation process at each PHC centre and moderating the meetings and seminars that were held as part of the implementation intervention.
- *Leadership engagement* [44], support and internal facilitation provided by the managers at each PHC centre.
- *Local champions* [45], support and internal facilitation provided by healthcare professionals at each PHC centre who were appointed as local PAP-S coordinators: two nurses and one physiotherapist at PHC-C1, one nurse, one physician and one physiotherapist at PHC-C2, and one nurse, one physician, one physiotherapist and one cognitive behavioural therapy (CBT) counsellor at PHC-C3.
- *Tailoring to local conditions* [46], producing local work routines tailored to each of the PHC centres’ conditions/context.
- *Educational outreach* [47], an ‘inspirational lecture’ concerning the health benefits of physical activity and an ‘educational lecture’ concerning the PAP-S method.
- *Audit and feedback* [48], the manager regularly providing statistics to the staff at each PHC centre on the number of PAP-S prescriptions.

The implementation strategies were applied by a step-wise procedure (Fig. 1). The focus for the researcher acting as external facilitator was to initiate local ownership of the change process through leadership engagement and local PAP-S coordinators. The PHC managers and the PAP-S coordinators were supposed to take the lead in the local activities concerning the implementation strategies: ‘tailoring to local conditions’ and ‘audit and feedback’.

The external facilitator was a key actor in the implementation intervention at the PHC centres, by being the contact for the PHC centres, attending the preparatory meetings and acting as seminar leader. The second author participated in all meetings and seminars as participatory observer. As depicted in Fig. 2, the implementation intervention started with three preparatory meetings held at each PHC centre: one with the PHC manager and two with the manager and the local PAP-S coordinators. At those meetings, the external facilitator provided the manager and the PAP-S coordinators with written material to use as a base for producing a draft for a local work routine for PAP-S. They were instructed to include the five specified core components of the PAP-S method in the local work routine but to tailor the application to the specific conditions at the PHC centre.

Then, three seminars were conducted with all healthcare staff and management at each PHC centre. At the first half-day seminar, an ‘inspirational lecture’ on the importance of health-promoting physical activity was provided by an external expert in the field, followed by an ‘educational lecture’ to provide basic understanding of the PAP-S method and its components, including easily applicable (hands-on) guidance on how to prescribe PAP-S. The PHC centre manager presented statistics from the patient record system on current number of PAP-S prescriptions at the PHC centre. The local PAP-S coordinators and PHC centre manager presented a draft for a local work routine for PAP-S based on the core components entailed in PAP-S and tailored to the specific conditions at the PHC centre. The draft was discussed at the seminar and further adjusted based on the comments of the healthcare staff.

The implementation intervention also included one-hour follow-up seminars at two months and six months after the first seminar. The follow-up seminars focused on discussion about the staffs’ experiences of using the PAP-S method in patient consultations, overcoming barriers and recognising facilitators in the use of the PAP-S method, and the feasibility of the written local work routine for PAP-S prescriptions. The local PAP-S coordinators were requested to revise the written local routine based on comments brought up at the follow-up seminars. They were also responsible for making the written routine visible to and known by all staff by posting it on the PHC centre’s internal digital platform.

### Data collection and analyses

#### Process evaluation of the implementation intervention

The implementation process was monitored by the second author who was the participatory observer during all meetings and seminars and took notes according to semi-structured protocols prepared prior to the study [40]. The observation protocols included space for notes covering operationalisation of the MRC framework [31] (Fig. 1), by describing aspects of the implementation intervention delivery in terms of ‘fidelity’ (i.e. delivery of planned implementation strategies within the planned time span and how they were applied), ‘dose delivered’ (i.e. the amount of meetings and seminars held at the PHC centres), ‘adaptations’ (i.e. deviations from the planned procedures of the implementation plan), ‘reach’ (i.e. the number/proportions of healthcare staff that attended meetings and seminars as part of the implementation intervention), as well as description of contextual factors. The protocols also had fields for notes on possible mechanisms of impact (i.e., implementation activities and contextual factors affecting the implementation process and outcome) [31]. As part of assessing contextual factors, the work-places’ culture in regard to readiness-to-change was operationalised as the healthcare professionals’ openness to novelty and expressions of willingness and engagement in undertaking work-place changes. The observations of the culture of the work-places were coded to one of the nine stages of preparedness for change according to the Community Readiness Model [49]. The model defines nine stages of community preparedness to mobilise in order to address a specific issue: 1) No Awareness, 2) Denial, 3) Vague awareness, 4) Preplanning, 5) Preparation, 6) Initiation, 7) Stabilisation, 8) Confirmation/Expansion, 9) Professionalization. The observation protocols were analysed using qualitative content analysis by a deductive approach [38]. The first and the second author performed the initial data analysis. By reading the observation protocols, meaning units, i.e. specific units of information relating to the process evaluation, were identified and coded according to predefined categories based on the MRC framework: contextual factors, implementation intervention delivery and process, implementation strategies, and mechanisms of impact. Then, all authors participated in discussing the coding and reviewed the preliminary interpretation until consensus was obtained.

#### Participants’ background characteristics, attitudes towards physical activity promotion, and PAP-S and experiences of the implementation intervention

Questionnaires were developed specifically for this study. The questions were not validated or tested in advance. The questions aimed to collect comprehensive information on the components and concepts relating to the PAP-S method and were based on the researchers’ knowledge of the PAP-S method and its theoretical foundations in concepts for inducing behaviour change. At baseline, the questionnaire contained questions on background characteristics (profession, year in profession, year working at the PHC centre), questions on attitudes and experiences of undertaking health promotion in patient encounters concerning insufficient physical activity, and questions on knowledge and experiences of using the PAP-S method to promote physical activity in health promotion consultations. At the follow-ups, the questionnaire contained the same questions but also questions on their experiences of participating in the implementation intervention. Questionnaire data were analysed using descriptive statistics and for differences over time (from baseline to follow-ups) by use of Chi-square test and Wilcoxon signed rank test [50]. Analyses were conducted using IBM SPSS 23.0.

Follow-up interviews were undertaken according to a semi-structured topic guide [51] prepared prior to the study. The topic guide contained open-ended questions covering:

- How was the implementation process perceived regarding delivery, acceptability, adherence and suitability in relation to context?
- Which components of the implementation intervention facilitated, respectively impeded using the PAP-S method?
- How was the use of the PAP-S method affected during and after the implementation intervention?

A purposeful sample of participants was invited to the interviews [40]. The sampling approach aimed at achieving participation of all healthcare managers, all local PAP-S coordinators and three healthcare professionals from each PHC centre. Thirteen participants accepted participation in interviews: three PHC centre managers, one former PHC centre manager, six of the healthcare professionals appointed as local PAP-S coordinators (two nurses from PHC-C1, one nurse, one physician and one physiotherapist from PHC-C2, and one physiotherapist from PHC-C3), and three healthcare professionals (one physician from PHC-C1, and one nurse and one physician from PHC-C3).

The third author conducted the follow-up interviews. The interviews lasted for 25–60 min and were carried out at the participant’s workplace with only the participant and the interviewer present, undisturbed by others. The interviews were audio-recorded, transcribed verbatim and analysed using qualitative content analysis by an inductive approach [38]. The first and the third author performed the initial data analysis. By reading the text, meaning units were identified, condensed and coded. Codes were grouped together into higher order subcategories and categories. All authors discussed the coding and reviewed the preliminary interpretation until consensus was obtained.

#### Outcome on PAP-S prescriptions

The number of written PAP-S prescriptions per month at each PHC centre was collected by aggregated statistics derived from the patient record system, covering two years before the implementation intervention and more than one year after the implementation intervention (January 2014 to December 2017).

#### Establishing rigour in data collection and the qualitative analyses

Since the study involved qualitative data collection from a small sample, measures were undertaken to ensure rigour and thus quality in the data collection [40, 52]. Qualitative and quantitative data collection served to complement each other and generate a systematic and rich data collection. We included a purposeful sample of three PHC centres that were representative to a Swedish healthcare context to order to interpret transferability. In addition, the observation protocols were theoretically based and operationalisation on the MRC framework which is a framework intended to guide process evaluations. The participating observer (second author) was well familiar with the MRC framework. To avoid investigator bias, a researcher (third author) who had not participated in the implementation intervention undertook the post-intervention interviews. The researcher performing the interviews was very experienced in undertaking data collection by interviews.

To ensure rigour in the interpretation of qualitative data, all of the researchers participated in the analyses of data collected by observations and interviews [40, 52]. All researchers had experience of qualitative content analysis of interviews. The first author had experience of content analysis of observations. All researchers had experience of working in healthcare. Four researchers had worked in PHC and were familiar with similar contexts to where the study took place. One of the researchers (last author) had extensive experience of implementation studies.

## Results

### Characteristics of participants and context

Table 2 provides background characteristics for the participants, as well as their self-assessed attitudes, self-efficacy for, and experiences of physical activity consultations, and of using the PAP-S method. In general, the healthcare professionals had a positive attitude towards undertaking health promotion concerning physical activity. At baseline, all participants (*n* = 76) considered it rather or very important for healthcare in general, and for them in their professional practice to promote physical activity. Still, only three participants (4%) reported that they used the PAP-S method to a rather large extent to promote physical activity in patient consultations. The observations indicated that at the start of the implementation intervention basic knowledge about the PAP-S methods and its components was lacking in the vast majority of healthcare staff and managers. This was also displayed in the baseline questionnaire where the majority (*n* = 45, 59%) reported that they had some general knowledge about the PAP-S method, but not about the specific components entailed in the PAP-S method.

**Table 2:** Baseline characteristics of the participating healthcare professionals and managers (*n* = 76) at the three PHC centres

	**PHC centre 1** (*n* = 41)	**PHC centre 2** (*n* = 17)^a^	**PHC centre 3** (*n* = 18)
	n (%)	n (%)	n (%)
Gender			
Female / Male	36 (87.8) / 5 (12.2)	11 (64.7) / 6 (35.3)	15 (83.3) / 3 (16.7)
Profession			
Nurse	19 (46.3)	6 (35.3)	5 (27.8)
Occupational therapist	3 (7.3)	0 (0.0)	0 (0.0)
PHC centre manager	1 (2.4)	2 (11.8)	1 (5.6)
Physician	8 (19.5)	4 (23.5)	5 (27.8)
Physiotherapist	7 (17.1)	1 (5.9)	2 (11.1)
Psychologist/CBT-therapist	3 (7.3)	0 (0.0)	1 (5.6)
Other (nurse aides)	0 (0.0)	2 (11.8)	2 (11.1)
Missing information	0 (0.0)	2 (11.8)	2 (11.1)
Years of working in the profession			
Less than 1 year	2 (4.9)	2 (11.8)	1 (5.6)
1–3 years	3 (7.3)	1 (5.9)	3 (16.7)
More than 3 years	36 (87.8)	12 (70.6)	12 (66.7)
Missing information	0 (0.0)	2 (11.8)	2 (11.1)
Years of working at the PHC centre			
Less than 1 year	9 (22.0)	4 (23.5)	3 (16.7)
1–3 years	6 (14.6)	0 (0.0)	5 (27.8)
More than 3 years	26 (63.4)	11 (64.7)	8 (44.4)
Missing information	0 (0.0)	2 (11.8)	2 (11.1)
How important is it that healthcare in general promote physical activity in patient consultations? (1–4)			
"Not at all important"	0 (0.0)	0 (0.0)	0 (0.0)
"Not especially important"	0 (0.0)	0 (0.0)	0 (0.0)
"Rather important"	8 (19.5)	5 (29.4)	0 (0.0)
"Very important"	33 (80.5)	10 (58.8)	16 (88.9)
Missing information	0 (0.0)	2 (11.8)	2 (11.1)
How important is it that you, in your profession practice, promote physical activity in patient consultations? (1–4)			
"Not at all important"	0 (0.0)	0 (0.0)	0 (0.0)
"Not especially important"	0 (0.0)	0 (0.0)	0 (0.0)
"Rather important"	15 (36.6)	6 (35.3)	5 (27.8)
"Very important"	26 (63.4)	9 (52.9)	11 (61.1)
Missing information	0 (0.0)	2 (11.8)	2 (11.1)
To what extent do you provide physical activity health promotion consultations in your current professional practice? (1–4)			
"To a very limited extent or not at all"	2 (4.9)	2 (11.8)	2 (11.1)
"To a rather small extent"	12 (29.3)	4 (23.5)	3 (16.7)
"To a rather large extent"	16 (39.0)	5 (29.4)	6 (33.3)
"To a very large extent"	11 (26.8)	3 (17.6)	5 (27.8)
Missing information	0 (0.0)	3 (17.6)	2 (11.1)
The Swedish Physical Activity on Prescription PAP-S, is a method in healthcare for promoting physical activity			
Are you familiar with the PAP-S method? (1–4)			
"No, I am not familiar with the PAP-S method"	2 (2.9)	0 (0.0)	0 (0.0)
"Yes, I have heard about the PAP-S method"	10 (24.4)	2 (11.8)	4 (22.2)
"Yes, I have some general knowledge about the PAP-S method, but not about its specific components"	26 (63.4)	8 (47.1)	11 (61.1)
"Yes, I am well familiar with the PAP-S method and its specific components"	3 (7.3)	4 (23.5)	1 (5.6)
Missing information	0 (0.0)	3 (17.6)	2 (11.1)
To what extent do you use the PAP-S method to promote physical activity in your professional practice? (1–4)			
"To a very limited extent or not at all"	23 (56.1)	6 (35.3)	9 (50.0)
"To a small extent"	15 (36.6)	6 (35.3)	4 (22.2)
"To a rather large extent"	2 (4.9)	0 (0.0)	1 (5.6)
"To a very large extent"	0 (0.0)	0 (0.0)	0 (0.0)
"I do not have patient consultations in my current position"	1 (2.4)	2 (11.8)	2 (11.1)
Missing information	5 (6.6)	3 (17.6)	2 (11.1)
Compared to your current situation, to what extent would you like to use the PAP-S method? (1–5)			
"To a much lesser extent than today or not at all"	0 (0.0)	0 (0.0)	0 (0.0)
"To a little lesser extent than today"	0 (0.0)	0 (0.0)	0 (0.0)
"To the same extent as today"	3 (7.3)	2 (11.8)	3 (16.7)
"To a little larger extent than today"	23 (56.1)	8 (47.1)	7 (38.9)
"To a much larger extent than today"	13 (31.7)	2 (11.8)	4 (22.2)
Missing information	2 (4.9)	5 (29.4)	4 (22.2)
	Median (IQR)	Median (IQR)	Median (IQR)
How confident do you feel about your ability to promote physical activity in patient consultations? (0–10)			
0 = "not at all confident", 10 = "very confident" (Missing information *n* = 5, 6.6%)	7 (5.5–8)	6 (3.75–9)	7 (4.25–8)
How confident do you feel about your ability to use the PAP-S method to promote physical activity in patient consultations? (0–10)			
0 = "not at all confident", 10 = "very confident" (Missing information *n* = 11, 14.5%)	3 (1–7)	4.5 (2.25–8.5)	4.5 (0.75–7)

^a^Including the PHC manager and two healthcare professionals at PHC-C2 that did not provide baseline questionnaire data. IQR: Interquartile range

### Process evaluation of the implementation intervention

An overview of the results of the coding of the observations and the interviews is provided in Appendix 1.

#### Implementation intervention delivery

The observations showed that the implementation intervention could not be undertaken as planned at all PHC centres. Nevertheless, the qualitative analysis of the observations indicated that ‘fidelity’ to the original implementation plan, i.e. the delivery of all planned implementation strategies within the planned time span, was as intended at all three PHC centres. The ‘dose delivered’ of the implementation intervention, i.e. the amount of meetings and seminars, was as intended, with the exception of one adaptation at PHC-C1, in that two additional meetings were held with the local PAP-S coordinators and the researcher to draft a local work routine. However, the implementation intervention did not ‘reach’ all staff at the PHC centres. Not all health professionals participated in the implementation intervention activities. About half of the 76 participants attended all three seminars: five participants did not attend the first seminar, about one third of the participants did not attend the second or third seminar. The clinical work had to go on at the PHC centres during seminars and made it difficult for all staff to participate in all activities.

#### Changes in context and participants’ attitudes and behaviour

Using the Community Readiness Model, the qualitative analysis of observation data indicated that the PHC-C1 workplace readiness-to-change, changed from stage 1–2 (i.e. ‘No Awareness’ – ‘Denial’) at baseline to stage 5 (i.e. ‘Preparation’) at the third seminar at the end of the implementation intervention. Quantitative analysis of questionnaires showed that the healthcare professionals increased confidence in their ability to use the PAP-S method from baseline (median: 3, IQR: 1–7) to the end of the implementation intervention (median: 6, IQR: 2.5–8) on an 11-point scale (*p* < 0.001). The observations showed that not all of the staff took an active part in the implementation process, in particular, the physicians and the manager participated less.

At PHC-C2 the workplace readiness-to-change was assessed to have changed from stage 4 (i.e. ‘Preplanning’) at baseline to 6 (i.e. ‘Initiation’) at the third seminar. The healthcare professionals increased confidence in their ability to use the PAP-S method to promote physical activity in patient consultations from baseline (median: 4.5, IQR: 2.25–8.5) to the end of the implementation intervention (median: 7.5, IQR: 3–8.75, *p* < 0.001). The observations showed that most of the healthcare professionals (and all professions) were engaged in the implementation process. All local PAP-S coordinators at PHC-C2 took an active part in the implementation process, but the PAP-S coordinator who had previous experience of such work, took a leading role and acted as the workplace local champion. However, she ended her employment at the PHC centre shortly after the end of the implementation intervention. Overall, there was a major staff turnover at PHC-C2 during this period of time, i.e. almost all nurses ended their employment and were replaced by new nurses. The first manager stopped working at the PHC-C2 immediately before the second seminar (in May 2016). The new manager started working one week before the third seminar (in September 2016).

At PHC-C3 the workplace readiness-to-change*,* changed from stage 3 (i.e. ‘Vague awareness) at baseline to 6 (i.e. ‘Initiation’) at the third seminar. The healthcare professionals increased confidence in their ability to use the PAP-S method to promote physical activity in patient consultations from baseline (median 4.5, IQR: 0.75–7) to the end of the implementation intervention (median: 7, IQR: 5–10, *p* < 0.001). The observations indicated that most of the healthcare professionals participated in meetings and seminars, and the manager took a leading role in the implementation process.

#### Participants’ experiences of the implementation process

At all three PHC centres, the participants were generally satisfied with the content of the implementation intervention. However, the healthcare professionals and the local PAP-S coordinators perceived that the time for preparation before the implementation intervention started and the duration of the implementation process were too short. All interviewed participants experienced that lack of time and resources affected the possibility to undertake implementation activities. They also regretted that the implementation process was activated at the PHC centres just before the summer holiday periods. This corresponds with the seminar observations showing that, although the preparatory meetings were in January and February and the first seminar was in March, it was not until the second seminar in May that the healthcare professionals became actively engaged in the implementation process.

In the follow-up questionnaire at the end of the implementation intervention, the actions rated most important to facilitate successful implementation of the PAP-S method were: ‘having the possibility to refer the prescription of PAP-S to a specialised function within the PHC centre or within some other part the healthcare organisation’ (*n* = 29, 38%); ‘having a routine for pre-planned and scheduled follow-up of the PAP-S prescription’ (*n* = 28, 37%); ‘having the PHC centre management taking active part in the intervention and actively supporting the use of PAP-S’ (*n* = 26, 34%); ‘having a special appointed function, "local PAP-S coordinator", at the PHC centre’ (*n* = 25, 33%); ‘having a written local work routine for PAP-S at the PHC centre’ (*n* = 22, 29%); ‘having the possibility to refer the prescription of PAP-S to a physiotherapist’ (*n* = 22, 29%). This corresponds with suggestions brought up by the healthcare professionals during the seminars at all three PHC centres. In particular, the healthcare professionals frequently mentioned that it was problematic to make time to carry out PAP-S prescription follow-ups and asked for a specialised function, e.g. the local PAP-S coordinator, who could take on follow-up of prescriptions.

#### Implementation strategies

##### External facilitation

The observations showed that external facilitation by the researcher (first author) was central to the implementation process. The external facilitation was supposed to initiate the implementation process and activate local ownership of the change process, but it had to actively push the process forward throughout the whole implementation intervention, which was confirmed by the interviews. The managers reported that they had received extensive support from the researcher, as expressed by one of the PHC managers in the follow-up interview: ‘*Nothing happened unless XX was present!*’ The observations showed that the researcher put much emphasis on increasing the involvement of the management and the local PAP-S coordinators in order to decrease the need for external facilitation.

##### Leadership engagement

Findings from the follow-up questionnaire underscored the perceived importance of leadership engagement, i.e. having the PHC centre management taking an active part in the implementation process and actively supporting the use of the PAP-S method. However, the healthcare professionals’ experience of leadership engagement and support from the management varied.

At PHC-C1, the observations and interviews disclosed that the healthcare professionals and the PAP-S coordinators perceived a lack of leadership involvement. The healthcare professionals expressed that the manager did not provide adequate support and was not engaged in the implementation activities. Apart from presenting statistics on PAP-S prescriptions at the three seminars, the manager at PHC-C1 took no active part in the implementation process. The manager felt that it was an obligation for the PHC centre to participate in the implementation intervention but expressed that it was hard to engage actively due to a heavy workload and the mental burden of having long-lasting staff shortages.

The observations showed that the first manager, who stopped working at the PHC-C2 immediately before the second seminar, contributed actively to the implementation process. The new manager, who started working one week before the third seminar, took no active part in the implementation process. The interviews indicated that the change of manager in PHC-C2, i.e. not having a manager between the second and third seminar, negatively affected the healthcare professionals’ experience of management support during the implementation intervention. The overall perception among the staff is illustrated by one PAP-S coordinator stating: ‘*We were without a manager during the entire* [implementation] *intervention*’.

At the PHC-C3, the observations showed that the manager took a leading role in the implementation activities right from the start by actively contributing to the writing of the local work routine, encouraging and supporting the staff and providing feed-back at the PHC centre’s workplace meetings on monthly numbers of PAP-S prescriptions. The manager stated that it was a great opportunity for the PHC centre to participate in a project focusing on implementation of the PAP-S methods in PHC. The healthcare staff expressed that their manager was very progressive regarding the undertaking of workplace improvements and engaged in encouraging and supporting the staff.

##### Local PAP-S coordinators

According to responses in the follow-up questionnaire and all of the interviewed participants, local PAP-S coordinators taking a leading role in the implementation process was perceived as important for successful implementation. However, several of the PAP-S coordinators experienced lack of time for the assignment. Some of them expressed that they were somewhat unsure about the job assignment, i.e. what tasks were connected to being a PAP-S coordinator. The observations also indicated that these PAP-S coordinators had little knowledge about the PAP-S method.

The local PAP-S coordinators at PHC-C1 had sparse knowledge regarding the PAP-S method, which was observed when they were asked to produce a draft of a local work routine for the PAP-S method, as they did not know what kind of information to include in the work routine.

At PHC-C2, the observations showed that one of the PAP-S coordinators took a leading role in the implementation process from the start, by acting as the workplace ‘local champion’ and supporting the rest of the staff. This PAP-S coordinator undertook several of the follow-ups of PAP-S prescriptions that the other healthcare professionals did not have time to do and provided feedback at the PHC centre’s workplace meetings regarding monthly numbers of PAP-S prescriptions. It is notable that prior to the implementation intervention, she had acted as local PAP-S coordinator at the PHC centre and was the participant who had the best knowledge of the PAP-S method.

The observations exposed that PHC-C3 had appointed four local PAP-S coordinators of which one was negative towards the PAP-S method, two were hesitant and one (a CBT counsellor) was positive owing to the fact that the PAP-S method corresponded to CBT principles. At the third and final seminar, one of the hesitant PAP-S coordinators expressed having figured out how to integrate the PAP-S method into the daily work routine and intended to continue to use the PAP-S method after the implementation intervention.

##### Tailoring to local conditions

The development of a local work routine for the PAP-S method was discussed at all seminars at all PHC centres. The development was however achieved in different ways and at different speeds at the PHC centres. All the interviewed participants mentioned that having a tailored local work routine was important for successful implementation. This was also confirmed by the follow-up questionnaire in which half of the participants deemed it important to have a written local work routine for the PAP-S method. It was considered especially important that the local work routine entailed procedures for teamwork and the possibility to refer patients to colleagues. It was deemed favourable if the work routine entailed the possibility of forwarding follow-up of PAP-S prescriptions to the local PAP-S coordinator.

At PHC-C1, the observations indicated that the local PAP-S coordinators had problems producing a draft for a work routine and requested two additional meetings with the researcher to obtain additional information on what to include in the work routine. After the second seminar, one of the local PAP-S coordinators took on the responsibility of writing a draft for a local work routine which was presented at the third seminar and agreed upon by the rest of the healthcare professionals.

At PHC-C2, a written local work routine was produced early in the implementation process. All PAP-S coordinators and the manager participated actively in drafting the local work routine, with the PAP-S coordinator who had previous experience of being PAP-S coordinator taking a leading role. In addition to the material that the external facilitator provided, they used material that had previously been handed out by the healthcare organisation’s health promotion department to all PHC centres. The local routine was presented to all staff and discussed at the seminars. However, at the follow-up interviews, the local PAP-S coordinators explained that there was a lack of adherence to the local work routine among the healthcare staff.

At the PHC-C3, the observations disclosed that the manager had a very firm opinion about the form and the content of the local work routine and took a leading role in the writing. For the third seminar, the manager, with some assistance from the local PAP-S coordinators, had produced a local work routine that was presented to all staff and put into action. The interviewed healthcare staff at PHC-C3 however expressed a lack of opportunity to participate in co-producing the local work routine as exemplified by the following quote: ‘*No-one asked us about what was important to get this thing working here at our PHC centre. I just have to follow the instructions. But it’s not that easy to follow instructions that you haven’t discussed and agreed upon in the work-team*’.

##### Educational outreach

At all three PHC centres, the observations during the ‘educational lecture’ revealed that very few among the health professionals had prior knowledge of the core components in the PAP-S method (apart from the written prescription), or how to undertake PAP-S prescriptions. Nor had the managers, as stated by one of the PHC centre managers: ‘*Oh, I didn’t know this! I should have had this information from the beginning, from the first time we started discussing conducting this intervention at our centre*’. The interviews with managers, local PAP-S coordinators and other healthcare staff confirmed that the participants appreciated that the ‘educational lecture’ provided basic knowledge of the PAP-S method and hands-on guidance on how to prescribe PAP-S. In contrast, the ‘inspirational lecture’ was perceived as somewhat redundant, because already prior to the implementation intervention all healthcare staff acknowledged the importance of promoting physical activity in patient consultations. The observations at seminars and the interviews clearly indicated that the participants needed more knowledge on ‘how to use’, not ‘why to use’ the PAP-S method. To quote one of the interviewed healthcare professionals: ‘*We already know why it is done, but we need more knowledge on how it is done*’.

##### Audit and feedback

The managers and/or local PAP-S coordinators were instructed to provide feedback to the staff at workplace meetings on numbers of PAP-S prescriptions undertaken at the PHC centres each month. At PHC-C1, the manager provided this feedback at the three seminars, not at the usual weekly workplace meetings. At PHC-C2, the local PAP-S coordinator, who took a leading role in the implementation process, and at PHC-C3, the manager, regularly provided statistics at workplace meetings on number of PAP-S prescriptions undertaken at the PHC centre. In the interviews, the local PAP-S coordinators and the healthcare staff expressed that they wanted more feedback on a regular basis of numbers of PAP-S prescriptions, and indicated that after the implementation intervention such feedback was absent. In the follow-up questionnaire, 32 (42%) of the participants considered it important to regularly receive feedback on the PHC centre's PAP-S prescriptions.

##### ***PAP-S prescriptions***

**Fig. 3:**
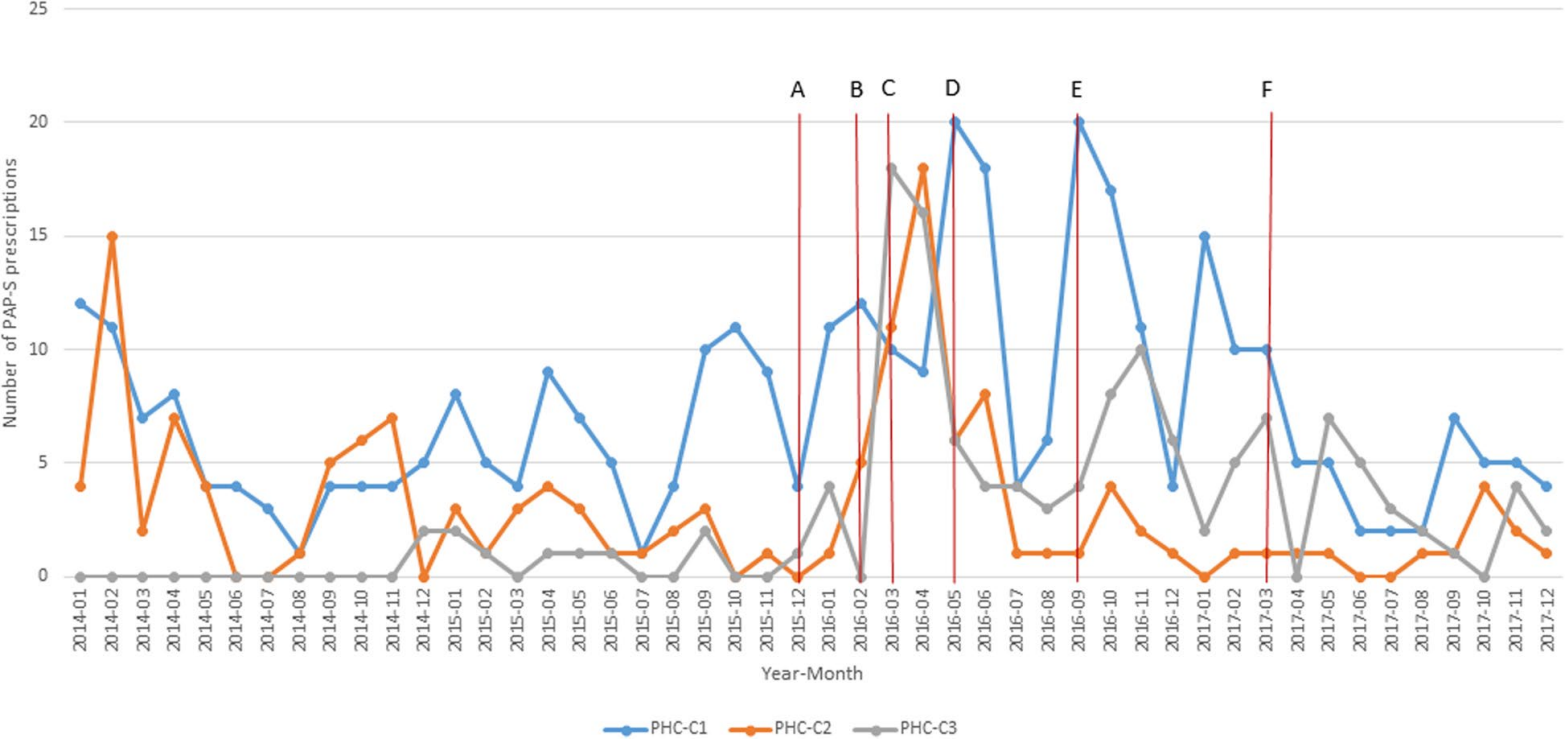
Number of PAP-S prescriptions at the three PHC centres from January 2014 to December 2017. Figure legend: Vertical lines indicating time-points (month) of contact between researchers and PHC centres as part of the implementation intervention and follow-ups: A) Contacts with the PHC centre managers, B) Meetings with the PHC centre managers and ‘local PAP-S coordinators’, C) First half-day seminar with all staff at each PHC centre, D) Second seminar with all staff at each PHC centre, E) Third seminar with all staff at each PHC centre, F) Follow-up by self-assessment questionnaire to the PHC staff at six months after end of the implementation intervention

As shown in Fig. 3, the trend is that the number of written PAP-S prescriptions increased at all PHC centres during the implementation intervention, with marked increases in the months when the three seminars took place. After the implementation intervention the number of PAP-S prescriptions decreased at all centres. Half a year after the implementation intervention, and at the time-point for the 15-month follow-up questionnaire, the number of written PAP-S prescriptions seemed to have returned to about the same level as before the implementation intervention, with the exception of PHC-C3, showing a trend possibly indicating that the number of prescriptions was larger than before the implementation intervention.

There were small favourable changes over time in the participants’ self-assessed use of the core components of the PAP-S method as measured by the follow-up questionnaires (Table 3). The number of participants who stated that they used the PAP-S method to a large or rather large extent increased from baseline (*n* = 3, 3.9%) to the end of the implementation intervention (*n* = 12, 15.8%) and remained greater at 6 months after the implementation intervention (*n* = 9, 11.8%) than prior to the implementation intervention.

**Table 3:** Baseline, 9 months and 15 months responses of the participants’ (*n* = 76) use of the PAP-S method

	Baseline	9-month follow-up	Chi-square^c^		15-month follow-up	Chi-square^c^	
	(*n* = 76)	(*n* = 76)	df	*p*-value	(*n* = 76)	df	*p*-value
**To what extent do you use the PAP-S method to promote physical activity in your professional practice?**			9	.001		9	< .001
"To a very limited extent or not at all" n(%)	38 (50.0)	23 (30.3)			18 (23.7)		
"To a small extent" n(%)	25 (32.9)	18 (23.7)			23 (30.3)		
"To a rather large extent" n(%)	3 (3.9)	12 (15.8)			8 (10.5)		
"To a very large extent" n(%)	0 (0.0)	0 (0.0)			1 (1.3)		
"I do not have patient consultations in my current position" n(%)	5 (6.6)	3 (3.9)			2 (2.6)		
Missing information^a^ n(%)	5 (6.6)	20 (26.3)			24 (31.6)		
**When you make a physical activity prescription, what of the following components are included:**							
A person-centred counselling talk on health promoting physical activity, for example by use of Motivational Interviewing (1–4)			6	.004		6	.021
"Never" n(%)	5 (6.6)	3 (3.9)			2 (2.6)		
"Seldom" n(%)	8 (10.5)	7 (9.2)			2 (2.6)		
"Often" n(%)	11 (14.5)	11 (14.5)			12 (15.8)		
"Always" n(%)	14 (18.4)	17 (22.4)			17 (22.4)		
Missing information^a^ n(%)	38 (50.0)	38 (50.0)			43 (56.6)		
Evidence-based diagnosis-specific physical activity advices based on FYSS^b^ (1–4)			9	.104		6	.002
"Never" n(%)	5 (6.6)	8 (10.5)			2 (2.6)		
"Seldom" n(%)	13 (17.1)	12 (15.8)			7 (9.2)		
"Often" n(%)	14 (18.4)	12 (15.8)			16 (21.1)		
"Always" n(%)	4 (5.3)	6 (7.9)			7 (9.2)		
Missing information^a^ n(%)	40 (52.6)	38 (50.0)			44 (57.9)		
A written prescription in the PAP-S form (1–4)			9	.017		6	.087
"Never" n(%)	3 (3.9)	4 (5.3)			0 (0.0)		
"Seldom" n(%)	11 (14.5)	9 (11.8)			8 (10.5)		
"Often" n(%)	7 (9.2)	8 (10.5)			5 (6.6)		
"Always" n(%)	18 (23.7)	18 (23.7)			21 (27.6)		
Missing information^a^ n(%)	37 (48.7)	37 (48.7)			42 (55.3)		
A routine for pre-planned and scheduled follow-up of the PAP-S prescription (1–4)			9	< .001		4	.002
"Never"	6 (7.9)	8 (10.5)			2 (2.6)		
"Seldom"	11 (14.5)	5 (6.6)			6 (7.9)		
"Often"	10 (13.2)	10 (13.2)			9 (11.8)		
"Always"	11 (14.5)	15 (19.7)			16 (21.1)		
Missing information^a^ n(%)	38 (50.0)	38 (50.0)			43 (56.6)		
Referral of the PAP-S prescription to a specialised function within the PHC centre or the healthcare organisation, e.g. a physiotherapist (1–4)			6	.119		9	.012
"Never" n(%)	7 (9.2)	11 (14.5)			8 (10.5)		
"Seldom" n(%)	10 (13.2)	11 (14.5)			8 (10.5)		
"Often" n(%)	19 (25.0)	12 (15.8)			14 (18.4)		
"Always" n(%)	1 (1.3)	4 (5.3)			3 (3.9)		
Missing information^a^ n(%)	39 (51.3)	38 (50.0)			43 (56.6)		
Referring patients to whom I have prescribed PAP-S, to an activity organiser outside healthcare (1–4)			4	.234		9	.001
"Never" n(%)	4 (5.3)	6 (7.9)			5 (6.6)		
"Seldom" n(%)	13 (17.1)	16 (21.1)			10 (13.2)		
"Often" n(%)	20 (26.3)	16 (21.1)			15 (19.7)		
"Always" n(%)	1 (1.3)	1 (1.3)			4 (5.3)		
Missing information^a^ n(%)	38 (50.0)	37 (48.7)			42 (55.3)		

^a^Missing information owing to not responding to the questionnaire or not responding to the question ^b^FYSS: the handbook “Physical Activity in the Prevention and Treatment of Disease"

^c^Chi-square test for analysis of within-group changes over time. Missing cases excluded from chi-square tests

## Discussion

This study provides an in-depth process evaluation of an implementation intervention that aimed to facilitate the use of the PAP-S method in PHC. The process evaluation points at the fact that four of the six implementation strategies appeared to have substantial impact on the implementation process: external facilitation; workplace leadership engagement; local champions; and educational outreach*.* However, the findings show that the implementation intervention was neither sufficient to produce a favourable long-term outcome on the number of PAP-S prescriptions, nor produce sustained change of the healthcare staffs’ behaviour concerning the use of the PAP-S method.

At baseline, the three PHC centres were at different stages of workplace readiness-to-change regarding using the PAP-S method as assessed by the Community Readiness Model. The workplaces’ readiness-to-change was favourably influenced during the implementation intervention, as was the participants’ confidence in their ability to use the PAP-S method to promote physical activity in patient consultations. Still, there were very modest effects on behaviour change concerning the participants’ use of the core component in the PAP-S method. Furthermore, the implementation intervention did not yield a maintained effect on number of PAP-S prescriptions. The monthly number showed a trend to increase during the 9-months implementation intervention, but after the intervention it decreased at all centres. Half a year after the implementation intervention, the number of written PAP-S prescriptions had returned to about the same as before the intervention, with the exception of PHC-C3.

Based on previous research [27, 28, 53, 54], we hypothesized that a multi-faceted implementation intervention entailing the use of several implementation strategies, would increase the likelihood of successful implementation. In line with the i-PARIHS framework on factors for successful implementation [30], this implementation intervention provided facilitation by use of an external facilitator supporting ‘leadership engagement’ and ‘local champions’. The managers and the PAP-S coordinators were supposed to take on and orchestrate the other implementation strategies. However, ‘external facilitation’ as a ‘catalyser’ igniting the implementation process, did not work as intended. External facilitation was essential for the implementation process throughout the whole implementation intervention, resulting in a trend indicating a favourable effect on the number of PAP-S prescriptions. However this effect faded when the external facilitation ended. It is possible that the managers’ and PAP-S coordinators’ lack of knowledge and experience of using the PAP-S method contributed to the lack of engagement in advocating the method, and was a mechanism that impeded sustainability of the implementation intervention. The fact that the PAP-S coordinator at PHC-C2 who had previous experience of being PAP-S coordinator, was the only one who took a leading role in the implementation process, acted as the workplace ‘local champion’ and supported the rest of the staff, speaks for this. Also, it became clear that the managers, apart from the manager at PHC-C3, were occupied by other issues perceived as more of a priority, such as shortage of staff, and did not have the capacity to act as change agents. Research has suggested that a central support unit within the healthcare organisation could be a way to build capacity for maintained implementation of new methods [55]. Capacity building refers to the provision of ongoing support to increase practitioners’ knowledge, skills, self-efficacy and motivation to implement evidence-based methods [56]. It is possible that such a central unit could have provided the necessary support for continued use of the PAP-S method.

‘Leadership engagement’ has been described as an important contextual factor for successful implementation [36]. The leadership’s behaviour is powerful for setting the workplace priorities and influencing the staff’s behaviour [44]. In this study, the participating healthcare staff underscored the importance of the PHC centre management actively supporting the use of the PAP-S method. However, leadership engagement was lacking at PHC-C1 and PHC-C2, while at PHC-C3 the manager firmly declared to the healthcare professionals that the PAP-S method should be used and actively contributed to all parts of the implementation process. It is likely that the leadership engagement at PHC-C3 was a mechanism that contributed to that the number of PAP-S prescriptions showed a trend to not decrease after the implementation intervention as much as at the other two PHC centres.

The implementation process was facilitated at PHC-C2 by one of the local PAP-S coordinators who took a leading role and acted as ‘local champion’. To be able to fulfil the role as local champion it is important to have in-depth knowledge of the method in question and to be recognised by the healthcare staff as the workplace expert who remind and support other staff [45]. The local PAP-S coordinators at the two other PHC centres did not take on this kind of leading role. However, the local PAP-S coordinator at PHC-C2 ended her employment shortly after the implementation intervention, which coincided with the number of PAP-S prescriptions decreasing.

The local work routine at PHC-C2 specified the tasks for the local PAP-S coordinator: being the workplace acknowledged expert on the PAP-S method and supporting the healthcare staff by undertaking follow-ups of PAP-S prescriptions. The follow-up questionnaires and the interviews pointed to that having the possibility to refer the prescription of PAP-S, or at least the follow-up of the prescription, to a specialised function would facilitate implementation. Thus, we suggest that the impact of a local PAP-S coordinator is strengthened if the local work routine entails the possibility of forwarding follow-ups of the PAP-S prescriptions to the local PAP-S coordinator in cases when the other healthcare staff lack time. Physiotherapists were often mentioned as suitable for the role as local PAP-S coordinators, owing to their expertise in prescribing physical activity and handling the most complex cases. However, the PAP-S method is intended for the wide range of patients presenting in PHC, not least for those with lifestyle related symptoms, e.g. metabolic syndrome [6, 41]. This suggests that professionals other than physiotherapists could be suitable for the role of local PAP-S coordinators.

The implementation strategy ‘educational outreach’*,* targeted both why use and how to use the PAP-S method. The analysis indicated that participants were already aware of why use. It is noticeable that, because of acknowledging the importance of promoting physical activity, the healthcare staff were motivated and prepared to change behaviour and adopt the PAP-S method. However, the findings suggest that education in how to use the PAP-S method is crucial. Most of the healthcare staff, including the managers and most of the local PAP-S coordinators, had sparse knowledge of the core components of the PAP-S method prior to the implementation intervention. The participants expressed a need of more competence in how to undertake PAP-S counselling and appreciated more knowledge and hands-on guidance on how to apply the PAP-S method. This is in line with a recent systematic review showing that multi-faceted implementation interventions that included educational strategies, reported positive effects on professional practice outcomes [57]. A lesson learned is that the educational lecture should have been introduced earlier in the implementation process, prior to when the managers and PAP-S coordinators started to draft the local work routines. We believe that would have helped them to produce the work routine and in a better way support the staff in the implementation process. In addition, it would have been beneficial for all PHC staff to get more extensive education, including hands-on practical training of the PAP-S method and feedback on performance, to develop and consolidate skills.

Fixen and colleagues emphasise that implementation should prioritise methods that fit as part of regular procedures [35]. The rationale for the implementation strategy ‘tailoring to local conditions’ was to ensure that the PAP-S method fitted as part of other procedures at the PHC centre by supporting the production of a written local work routine for PAP-S at each PHC centre. Indeed, having a work routine tailored to local conditions was considered important by the participants. However, this was compromised by insufficient knowledge of the PAP-S method among the participants prior to the implementation intervention.

In regard to the implementation strategy* ‘*audit and feed-back’*,* the participants considered it important to get regular statistics of the PHC centre's PAP-S prescriptions. Feedback on accomplishments has been reported as an effective strategy to promote behaviour change in individuals [48, 58]. Lack of such feedback might have contributed to the lack of sustained behaviour change among the PHC staff concerning the use of the PAP-S method.

### Strengths and limitations

It is a strength of the study that the implementation intervention was based on best available evidence for supporting implementation [27, 29, 33]. The extensive data collection by multiple methods is also a major strength of this study. We considered a mixed methods approach to be appropriate for the process evaluation of this implementation intervention [31]. Collecting data by multiple sources served as methodological triangulation and strengthened the study's credibility [37, 40]. It is a strength of the study that the qualitative data collection involved observations and not only relied on interviews, since observations, in contrast to interviews, consider the individuals’ behaviour and actions, not only their spoken words.

A potential limitation of the study is that the first author had the external facilitator role and participated at all meetings and seminars at the PHC centres. To avoid bias due to this fact, an observer (second author) was present at all meetings and responsible for note-taking. The observer also administered the questionnaires to participants. In addition, the interviews with participants were undertaken by another researcher (third author). Further, all authors have been involved in reviewing the analyses and discussing the interpretation of the findings.

It is also a limitation of the study that the only data available from the patient record system on the core components of the PAP-S method was the number of written PAP-S prescriptions. Data on the other core components was not available from the patient record system, which hampered the possibility to evaluate the quality of the PHC staffs’ clinical performance and fidelity to the PAP-S method. Instead, we collected data on the use of the other core components in the PAP-S method by self-assessment questionnaires. The questionnaires were developed specifically for the study but unfortunately not validated and tested in advance. It is a limitation that the findings related to these core components in the PAP-S method relied on self-reported data from non-validated questionnaires.

Another limitation of the study is that a large number of participants did not respond to the follow-up questionnaire. A reason for this was that several participants did not attend the third seminar. Also, we did not succeed in interviewing the purposefully selected sample of participants after the implementation intervention. One of the local PAP-S coordinators had stopped working at the PHC centre, one was long-term sick-listed, and five of the local PAP-S coordinators and several among the healthcare professionals declined to allot time for an interview citing heavy workloads. However, the data collection by the observations during the implementation intervention, i.e. at preparatory meetings and seminar, provided rich material. Through the analyses of the observations and complementary information from the other data sources, we are confident that the research questions have been covered.

The small sample size consisting of only three PHC centres and the qualitative methods used for data collection, are limitations that prevents interpretation of causality and wider generalisability of the findings. Future studies involving a larger number of PHC centres, preferably using a cluster-randomized design, as well as tested and validated instruments for data collection, are needed. However, the purposeful sampling approach aimed at including PHC centres that displayed common characteristics of Swedish PHC centres, strengthens the possibility of providing useful information for other PHC centres and for the design of future large-scale implementation studies.

## Conclusion

The implementation intervention was not sufficient to produce sustained change of the healthcare staff’s behaviour concerning the use of the PAP-S method, nor did it achieve a favourable long-term outcome on the number of PAP-S prescriptions. The healthcare staffs’ sparse knowledge of the PAP-S method prior to the implementation intervention hampered the implementation. More hands-on education in how to use the PAP-S method introduced early in the implementation process is imperative for successful implementation of the PAP-S method. The findings also suggest that the implementation process was too short and that committed workplace management and local PAP-S coordinators taking a leading role and acting as local champions need to be firmly established before an external facilitator withdraws.

### Supplementary Information

**Additional file 1.**

## Data Availability

The datasets generated and analysed during the current study are not publicly available as they consist of detailed descriptions from observations and quotes by the interview subjects that might contain personal information, which could reveal the identity of individuals. Datasets on coded questionnaire data are available from the corresponding author upon reasonable request.

## Abbreviations

PAP-S: The Swedish Physical Activity on Prescription
PHC: Primary healthcare
