# Supplementary material for: Process evaluation of an implementation intervention to facilitate the use of the Swedish Physical Activity on Prescription in primary healthcare
Source: BMC Health Serv Res. 2023 Sep 15;23:996. doi: 10.1186/s12913-023-09974-8 (PMC10504760; doi:10.1186/s12913-023-09974-8)
Supplement: Supplementary file 1 — Additional file 1. [file 12913_2023_9974_MOESM1_ESM.docx]

**Additional file 1:**

| **Title**: Overview of the coding of the observations during the implementation intervention and the interviews undertaken after the implementation intervention to the categories: contextual factors, implementation intervention delivery and process, implementation strategies and mechanisms of impact. | | |
| --- | --- | --- |
| **Categories** | **Subcategories** | **Description of content in the subcategories** |
| Contextual factors | A general positive attitude among healthcare staff and management towards undertaking health promotion in regard to physical activity in patient encounters | The healthcare staff had a positive attitude towards undertaking health promotion in regard to physical activity in patient encounters, but the vast majority were reluctant towards using the PAP-S method for that purpose |
|  |  |  |
|  | Varying level of knowledge about the PAP-S method among the staff at the three PHC | The managers and the local PAP-S coordinators expressed that the knowledge about the PAP-S method varied among the healthcare staff. The observations revealed that the knowledge of how to use the PAP-S method was sparse among the vast majority of the staff. |
|  |  |  |
|  | Support and commitment from the management for undertaking work-place improvement efforts varied | The healthcare staff and the local PAP-S coordinators in one of three PHC centres perceived that their manager were interested and supportive during the implementation intervention process, whilst the vast majority of the staff at the other two PHC centres did not |
|  |  | Change of manager during the implementation intervention at one of three PHC centres (PHC-C2) affected the leadership engagement in the implementation intervention negatively, since the new manager was less interested in implementing the PAP-S method than the former manager |
|  |  | One of the managers expressed that the implementation intervention was an opportunity for the PHC centre to participate in the implementation of PAP-S and another of the managers expressed that it was an obligation for the PHC centre to participate in the implementation intervention |
|  | Variation in staffing and staff shortages before and during the intervention process | At two of the PHC centres (PHC-C1 and PHC-C2) the healthcare staff and the managers expressed that they suffered from staff shortages, both physicians, nurses and physiotherapists, which negatively affected both capacity and motivation to engage in implementation of new methods and routines |
|  |  | At one of the PHC centres (PHC-C3) the staff had been a stable since 2003 and there were no staff shortages |
| Implementation intervention delivery and process | Lack of opportunity to participate in planning the implementation | The healthcare staff perceived lack of opportunity to participate in planning the implementation intervention |
|  | Lack of time and resources to undertake the intervention | The healthcare staff, the managers and the local PAP-S coordinators experienced lack of time to participate in the implementation intervention process |
|  |  | The managers and the local PAP-S coordinators experienced that the time for the implementation intervention process was too short |
|  |  | The healthcare staff and the local PAP-S coordinators experienced that the time for preparation before the implementation intervention started was too short |
|  |  | All participants experienced that it was unfortunate that they perceived the implementation intervention started just before the summer and the holiday period |
| Implementation strategies | External facilitator - Researcher as ‘catalyser’ | The importance of the researcher was perceived as much more than a ‘catalyser’ (i.e. an external ingredient activating the implementation process): the researcher was an essential facilitator for carrying out the implementation intervention throughout the whole process |
|  |  | The managers perceived that they received plentiful of support from the researcher, and that it was essential for carrying out the implementation intervention |
|  |  | When the researcher was not present at the PHC centre nothing happened: the local PAP-S coordinators and the manager did not know what to do without the researcher’s clear instructions. |
|  | Leadership engagement | The healthcare staff and the local PAP-S coordinators in two of three PHC centres perceived lack of leadership involvement in the implementation process |
|  |  | The healthcare staff and the local PAP-S coordinators in two of three PHC centres perceived lacking leadership engagement in undertaking implementation activities |
|  |  | The manager at one of the PHC centres was very actively contributing to implementation activities in the implementation intervention |
|  | Local PAP-S coordinator ‘Local champions’ | All interviewed participants agreed that local PAP-S coordinators that take leading roles in the implementation are very important for a successful implementation of the PAP-S method. Important tasks for a successful PAP-S coordinator: being the workplace acknowledged expert on the PAP-S method who remind and support the rest of the staff; responsible for updating the local work routine; undertake follow-ups of PAP-S prescription when the other healthcare staff lack time; and provide feed-back at the PHC centre’s workplace meetings of monthly statistics on numbers of PAP-S prescriptions. |
|  |  | Several of the local PAP-S coordinators experienced lack of time for the assignment, for example finding time for meeting and collaborating to prepare the draft of the local routines for the PAP-S method. |
|  |  | Some of the local PAP-S coordinators were unsure about the job assignment, i.e. what tasks were connected to being a PAP-S coordinator |
|  |  | One of the local PAP-S coordinator (at PHC-C2) had previous experience of being PAP-S coordinator and was the only one of the PAP-S coordinators who fully took on the role as ‘local champion’. She ended her employment shortly after the end of the implementation intervention and no one else replaced her in the role. |
|  | Tailoring to local conditions | To have a local work routine tailored to the local conditions was important and facilitated undertaking the implementation intervention |
|  |  | The healthcare staff perceived that it was especially important that the local work routine entailed routines for teamwork and the possibility to refer patients to colleagues |
|  |  | The healthcare staff at one PHC centre perceived they did not participated in planning the local tailoring of the implementation intervention i.e. in producing the written local work routine |
|  |  | The local PAP-S coordinators and manager at one PHC centre (PHC-C2), who early in the implementation process produced a written local work routine, experienced insufficient adherence to the local written work routine among the healthcare staff |
|  | Educational outreach | It was favourable for implementation that all staff got the same information at the same time |
|  |  | It was perceived as difficult for the staff to participate in all of the educational activities, i.e. at the seminars, due to need to maintain usual work tasks at the PHC centre |
|  |  | The educational lecture was appreciated - The inspirational lecture was perceived redundant |
|  |  | The healthcare staff expressed a need for more competence concerning hands-on how to do PAP-S counselling and wanted more knowledge about "how to use", not "why to use" |
|  | Audit and feedback | The staff at two of the PHC centre (PHC-C2 and PHC-C3) got feed-back on monthly PAP-S prescriptions at work-place meeting during the implementation intervention, while the staff at one of the PHC centre (PHC-C1) did not |
|  |  | The healthcare staff wanted to have feed-back on numbers of PAP-S prescriptions on a regular basis, but experienced lack of such feedback during and after the implementation intervention |
| Mechanisms of impact | External facilitator - Researcher as ‘catalyser’ | The importance of the researcher was perceived as much more than a ‘catalyser’ (i.e. an external ingredient activating the implementation process): the researcher was an essential facilitator for carrying out the implementation intervention throughout the whole process. |
|  |  | The managers perceived that they received plentiful of support from the researcher, and that it was essential for carrying out the implementation intervention |
|  | Leadership engagement by committed and firm workplace management | A committed and firm workplace management that emphasized the urgency of PAP-S, decided and firmly declared to the staff that the PAP-S method would be used, and actively encouraged and supported the staff, facilitated implementation of the PAP-S method and was crucial for maintained use of the PAP-S method after the implementation intervention |
|  | Local PAP-S coordinator taking an active leading role | Having local PAP-S coordinators that take a leading role is very important for a successful implementation of the PAP-S method. The implementation intervention was more easily undertaken at the PHC centre (PHC-C2) where a local PAP-S coordinator took a leading role. Important tasks for a PAP-S coordinator: being the workplace acknowledged expert on the PAP-S method who reminds and supports the rest of the staff; responsible for updating the local work routine; undertake follow-ups of PAP-S prescription when the other healthcare staff lack time; and provide feed-back at the PHC centre’s workplace meetings of statistics on monthly numbers of PAP-S prescriptions. |
|  |  | This mechanism of impact was strengthened if the local work routine entailed the possibility of forwarding follow-up of PAP-S prescriptions to the local PAP-S coordinator in cases when the other healthcare staff experience lack of time |
|  | Educational outreach, by education in how to use the PAP-S method | Providing education in ‘how to use’ the PAP-S method for all employees was imperative, since the majority of the staff, including the managers and some of the local PAP-S coordinators, had very little knowledge about the PAP-S method prior to the implementation intervention. All participants expressed a need for more competence in how to undertake PAP-S counselling and wanted more hands-on knowledge in ‘how to use’. In contrast, The observations and interviews clearly showed that they were already fully aware of and positive to the ‘why to use’. |
